# Do differences in life‐history traits and the timing of peak mating activity between host‐associated populations of *Chilo suppressalis* have a genetic basis?

**DOI:** 10.1002/ece3.2227

**Published:** 2016-06-08

**Authors:** Wei‐Li Quan, Wen Liu, Rui‐Qi Zhou, Sundas Rana Qureshi, Nan Ding, Wei‐Hua Ma, Chao‐Liang Lei, Xiao‐Ping Wang

**Affiliations:** ^1^ Hubei Insect Resources Utilization and Sustainable Pest Management Key LaboratoryCollege of Plant Science and TechnologyHuazhong Agricultural UniversityWuhan430070China

**Keywords:** *Chilo suppressalis*, host plant, host race, life‐history trait, mating rhythm, phenotypic plasticity

## Abstract

The development of host races, genetically distinct populations of the same species with different hosts, is considered to be the initial stage of ecological speciation. Ecological and biological differences consistent with host race formation have been reported between water‐oat and rice‐associated populations of *Chilo suppressalis*. In order to confirm whether these differences have a genetic basis, we conducted experiments to determine the extent to which various life‐history traits and the time of peak mating activity of these populations were influenced by the species of host plant larvae were raised on. Individuals from each population were reared for three consecutive generations on either water‐oat fruit pulp or rice seedlings. Descendants of both populations had higher larval survival rates, shorter larval developmental periods, higher pupal weight, and longer adult forewings, when reared on water‐oats than when reared on rice. The time of peak of mating activity differed between the descendants of each population, irrespective of whether they were raised on water‐oats or rice. These results indicate that although some life‐history traits of host‐associated populations of *C. suppressalis* are influenced by the host plant larvae are raised on, time of peak mating activity is not. Because it is a stable, objective, phenotypic trait, further research on difference in the time of peak mating activity between host‐associated populations of *C. suppressalis* should be conducted to clarify the mechanism responsible for host race formation in this species.

## Introduction

Host race formation is thought to be the first step in the process of ecological speciation responsible for much of the diversity of phytophagous, parasitic invertebrates, and even some vertebrates (Via 2001). Indeed, the genetic divergence of host races through their adaptation to different host plants is considered to have contributed to insects becoming the most diverse multicellular animal taxon on Earth (Dres and Mallet 2002; Funk et al. 2002). Host races are defined as sympatric parasite populations that have different hosts, appreciable gene flow, and a degree of genetic divergence (Dres and Mallet 2002). Host races generally differ in life‐history traits such as survival rate, developmental period, body size and mating behavior (Feder and Filchak 1999; Nosil et al. 2002; Calcagno et al. 2007; Schöfl et al. 2009; Ragland et al. 2011). However, such differences can reflect phenotypic plasticity to different hosts rather than genetic divergence between host races (Dres and Mallet 2002; Gorur et al. 2005; Li and Ó Foighil 2012).

The striped stem borer, *Chilo suppressalis* (Lepidoptera: Crambidae), is an important insect pest of gramineous plants, particularly rice *Oryza sativa* (Linnaeus) and water‐oats *Zizania latifolia* (Turcz), in Asia (Maki and Yamashita 1956; Samudra et al. 2002; Hou et al. 2009). Indeed, although they parasitize other plants, *C. suppressalis* can only complete its life cycle on either rice or water‐oats (Maki and Yamashita 1956; Takasaki et al. 1969; Jiang et al. 2015). Rice and water‐oat crops are usually planted in a mosaic fashion in adjacent fields, or under a crop rotation system in the same field (Tsuchida and Ichihashi 1995; Matsukura et al. 2009). *C. suppressalis* has three generations a year in rice and water‐oat fields in Zhejiang Province, China (Qian et al. 2007), and two generations a year in most parts of Japan (Matsukura et al. 2009). Consistent ecological and biological differences between *C. suppressalis* populations associated with either rice or water‐oats suggests the existence of distinct host races associated with these crops (Matsukura et al. 2006, 2009; Ueno et al. 2006).

*Chilo suppressalis* that feed on water‐oats have larger bodied larvae, pupae and adults than those that feed on rice (Maki and Yamashita 1956; Tsuchida and Ichihashi 1995; Matsukura et al. 2006; Ding et al. 2013). The seasonal peak of emergence also differs between rice and water‐oat crops (Maki and Yamashita 1956; Tsuchida and Ichihashi 1995; Matsukura et al. 2009). Furthermore, in rice crops, courtship and mating begin in the first half of the scotophase, whereas in water‐oat crops these begin in the second half (Konno and Tanaka 1996; Samudra et al. 2002; Ishiguro et al. 2006; Ueno et al. 2006). These differences in morphology, temporal abundance, and mating behavior are consistent with the existence of host races (Matsukura et al. 2006, 2009; Ueno et al. 2006). However, they could also simply be the result of phenotypic plasticity to different host plants. Host plants have profound effects on the life‐history traits of many insects (Landolt and Phillips 1997; Gorur et al. 2005; Geiselhardt et al. 2012; Havens and Etges 2013; Etges and de Oliveira 2014; Gould and Wilson 2015). For example, larval rearing substrates can induce premating isolation in *Drosophila* (Havens and Etges 2013) and in the leaf beetle *Phaedon cochleariae* (Geiselhardt et al. 2012). In *C. suppressalis*, the differences in body size between individuals from rice or water‐oat crops are thought to be host plant effects rather than genetic (Maki and Yamashita 1956). However, it is also possible that differences in life‐history traits, including body size, have some genetic basis. Similarly, although differences in the timing of peak mating activity between *C. suppressalis* from rice and water‐oat crops are thought to have a genetic basis (Samudra et al. 2002), it is unclear to what extent this is influenced by host plant.

In order to determine whether the observed differences between water‐oat and rice‐associated populations of *C. suppressalis* have a genetic basis, or are due to phenotypic plasticity to different host plants, we experimentally reared individuals from each population on either host plant for three generations and compared selected life‐history traits of each putative host race and host plant combination. Although several life‐history characteristics were affected by host plant, the timing of peak mating activity was not. This suggests that the latter has a genetic basis and therefore supports the existence of distinct host races of *C. suppressalis*.

## Materials and Methods

### Experimental insects

*Chilo suppressalis* used in experiments were descended from overwintering larvae collected from either water‐oat fields (*n* ≈ 1000) at the Wuhan Vegetable Research Institute (114°16′E, 30°28′N), or rice fields (*n* ≈ 1500) in Hexian Village (113°57′E, 30°29′N), Wuhan, China. These larvae were mass‐reared in an insectarium at 28 ± 1°C, 80% relative humidity, under a photoperiod of 15‐h light:9‐h dark (LD 15:9 h). Light intensity during the photophase was approximately 2000–2500 lx. Larvae from the water‐oat population (W) were reared on fresh water‐oat fruit pulp (w) (the water‐oat of cultivar Ejiao 2) according to the method described by Meng et al. (2003) and those from the rice population (R) were reared on rice seedlings (r) (the rice of cultivar Fengyuanyou 299) according to the method described by Sato (1964). The next three generations of W and R larvae reared under the above conditions were used in subsequent experiments.

### Experimental design

**Figure 1:**
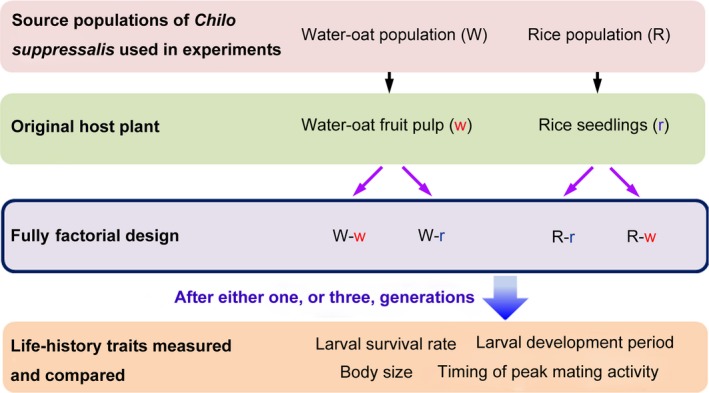
Schematic diagram of the fully factorial experimental design used in this study. *Chilo suppressalis* from water‐oat (W) and rice fields (R) were reared on either water‐oats (w) or rice (r) for three continuous generations to examine the effects of host plant and population origin on the life‐history traits of the G1 and G3 generations. There were two treatment groups comprised of W individuals reared on rice seedlings (W‐r) and R individuals reared on water‐oat fruit pulp (R‐w). The corresponding control groups were W individuals reared on water‐oat fruit pulp (W‐w) and R individuals reared on rice seedlings (R‐r).

A fully factorial experimental design was used; two groups of W larvae were reared on either rice seedlings (W‐r) or water‐oats (W‐w), and two groups of R larvae were reared on either water‐oats (R‐w) or rice seedlings (R‐r) (Fig. 1). Because *C. suppressalis* typically completes three generations a year in the wild (Qian et al. 2007), these treatment groups were maintained under these conditions for three successive generations. The effects of different host plants on life‐history traits and the timing of peak mating activity were observed and compared between the first (G1) and third (G3), generations of each treatment.

### Measurement of life‐history traits

Groups of eighty newly hatched larvae were randomly selected from egg masses laid by different females of the same putative host race, transferred into transparent glass pots (diameter 8 cm*height 12 cm) and reared on either water‐oats or rice seedlings. Each treatment group had three replicates. Water‐oats were replaced at 5‐day intervals before the third instar and at 1‐day intervals after the third instar. Rice seedlings were replaced at 10‐day intervals before the third instar and 5‐day intervals after the third instar. Although some larvae in each treatment group died during the course of experiment there was no epidemic disease. Larval survival rates and developmental periods were recorded for each treatment (Chen et al. 2003). New pupae were transferred daily to a transparent plastic box (diameter 15 cm*height 6 cm) and their sex and weight determined. These boxes were checked daily until eclosion, after which the forewing length of the newly adults was measured (Matsukura et al. 2006). Additional insects were reared at the same density and treatment conditions in another 17–21 boxes in order to obtain sufficient adults for the observation of mating behavior and sustain the captive population.

### Observation of the timing of peak mating activity

Mating activity was observed at 28 ± 1°C, 80 ± 10% relative humidity and LD 15: 9 h. About 60% of all mating in *C. suppressalis* is performed in the 24 h immediately after eclosion (Kanno 1978; Konno and Tanaka 1996). Therefore, we restricted observation of mating behavior to 1‐day‐old adults. Adult pairs comprised of a newly emerged 1‐day‐old virgin female and newly emerged 1‐day‐old virgin male from each treatment group were randomly selected and placed in an inverted, transparent plastic cup (200 mL) on an uncovered plastic petri dish (diameter 9 cm) containing cotton soaked in honey water (10% volume/volume). The time of the start of mating in each pair was recorded at 30‐min intervals during the scotophase under a flashlight covered with red cloth (Ishiguro et al. 2006; Ueno et al. 2006). The light intensity measured at a distance of 20 cm from the flashlight was less than 50 lx during scotophase and did not interfere with adult's normal activity (Kanno 1980).

### Data analysis

All analyses were performed in SPSS 11.5 (SPSS Inc., Chicago, IL, USA). Differences in larval survival rate, developmental period, and pupal and adult size were analyzed using a two‐way analysis of variance (ANOVA), with putative host race and host plant as the main factors. Levene's tests and QQ plots were used to confirm homogeneity of variance and normality of the data, respectively. Survival rates were normalized with an arcsine transformation before statistical analysis.

Under the ANOVA model, a significant host plant effect would be evidence of phenotypic plasticity, whereas a significant population effect would be evidence of genetic divergence between putative host races (Torres‐Vila and Rodríguez‐Molina 2013). A significant population–host plant interaction would, however, suggest that the effects of any genetic differences between putative host races are dependent to some extent on host plant. When a significant host plant, or population, effect was detected in a measured parameter, Tukey's HSD test was used to assess the significance of differences in these parameters between populations reared on the same host plant. The significance of differences in peak mating activity between different treatments, and between different generations of the same treatment, was assessed with a chi‐square test (Ueno et al. 2006); α = 0.05 for all tests.

## Results

### Larval survival on different host plants

**Figure 2:**
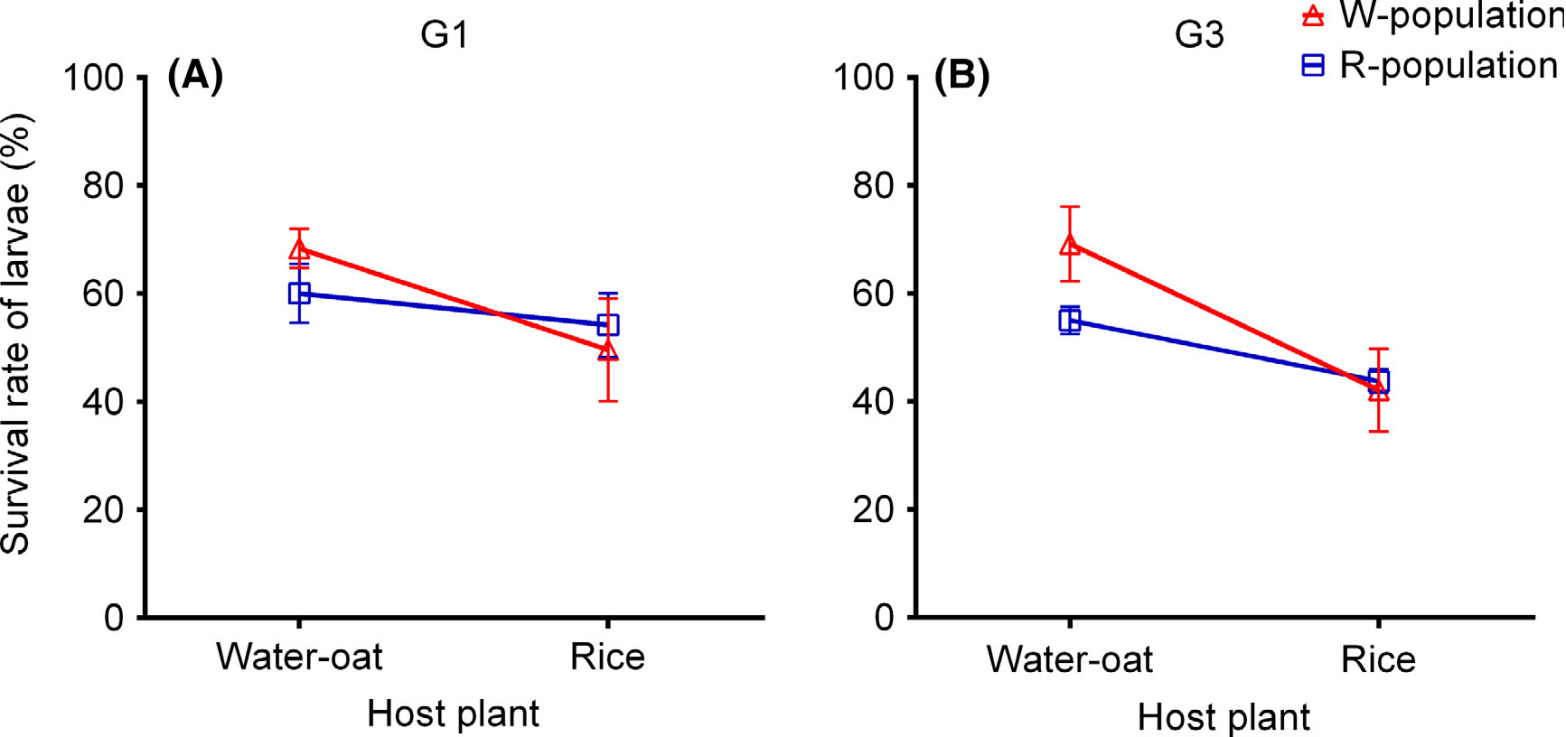
Larval survival rates of (A) the G1, and (B) G3 generations, of putative rice (R) and water‐oat (W) host races of *Chilo suppressalis* fed on either water‐oats or rice. □ = R, ▵ = W (see Table 1 for statistical analysis).

Host plant had a significant effect on larval survival (Table 1). Population origin did not have significant effect on survival of the G1 generation but did have a weak effect on the G3 generation (Table 1). There was also a significant population–host interaction effect on larval survival in the G3 generation (Table 1). The survival rates of W‐r larvae significantly decreased (Tukey's HSD: G1, *P *<* *0.05; G3, *P *<* *0.05), whereas those of R‐w larvae increased (Fig. 2A,B). There was a significant difference in larval survival between the G3 generations of the W‐w and R‐w treatment groups (Tukey's HSD: G3, *P *<* *0.05) (Fig. 2B).

**Table 1:** Results of two‐way ANOVA analyzing the effects of putative host race and host plant on selected life‐history traits of *Chilo suppressalis*. Factors were regarded as fixed effects, and each factor (host plant and population) had two levels (water‐oats and rice). df = degrees of freedom, MS = mean squares, *F *= Fisher's *F*‐statistic. Values in bold are significant at *P < *0.05. (see Fig. 1 for details of experimental design)

Life‐history traits	Factor	First generation				Third generation			
		df	MS	*F*	*P*	df	MS	*F*	*P*
Survival rate of larvae	Population (P)	1	10.547	0.252	0.629	1	117.188	4.018	0.080
	Host plant (H)	1	453.255	10.844	**0.011**	1	1102.083	37.786	**0.000**
	P*H	1	125.130	2.994	0.122	1	188.021	6.446	**0.035**
	Error	8	41.797			8	29.167		
Developmental period of larvae	Population (P)	1	14.890	0.831	0.362	1	0.229	0.012	0.912
	Host plant (H)	1	9504.649	530.349	**0.000**	1	9180.202	486.946	**0.000**
	P*H	1	1724.677	96.235	**0.000**	1	1200.772	63.693	**0.000**
	Error	553	17.921			500	18.853		
Body weight of female pupae	Population (P)	1	2131.860	26.404	**0.000**	1	2074.719	28.973	**0.000**
	Host plant (H)	1	10950.881	135.632	**0.000**	1	8505.300	118.773	**0.000**
	P*H	1	0.811	0.010	0.920	1	44.211	0.617	0.433
	Error	134	80.740			126	71.610		
Body weight of male pupae	Population (P)	1	1609.808	36.898	**0.000**	1	863.292	25.113	**0.000**
	Host plant (H)	1	1642.328	37.644	**0.000**	1	1401.534	40.770	**0.000**
	P*H	1	42.927	0.984	0.323	1	0.580	0.017	0.897
	Error	137	43.628			129	34.376		
Length of female forewing	Population (P)	1	3.969	14.702	**0.000**	1	20.660	76.088	**0.000**
	Host plant (H)	1	26.485	98.106	**0.000**	1	30.748	113.239	**0.000**
	P*H	1	0.007	0.026	0.873	1	1.140	4.198	**0.042**
	Error	122	0.270			130	0.272		
Length of male forewing	Population (P)	1	8.517	85.271	**0.000**	1	10.900	74.458	**0.000**
	Host plant (H)	1	5.364	53.708	**0.000**	1	9.270	63.323	**0.000**
	P*H	1	0.000	0.002	0.967	1	0.003	0.021	0.885
	Error	123	0.100			130	0.146		

### Effect of host plant on larval development

**Figure 3:**
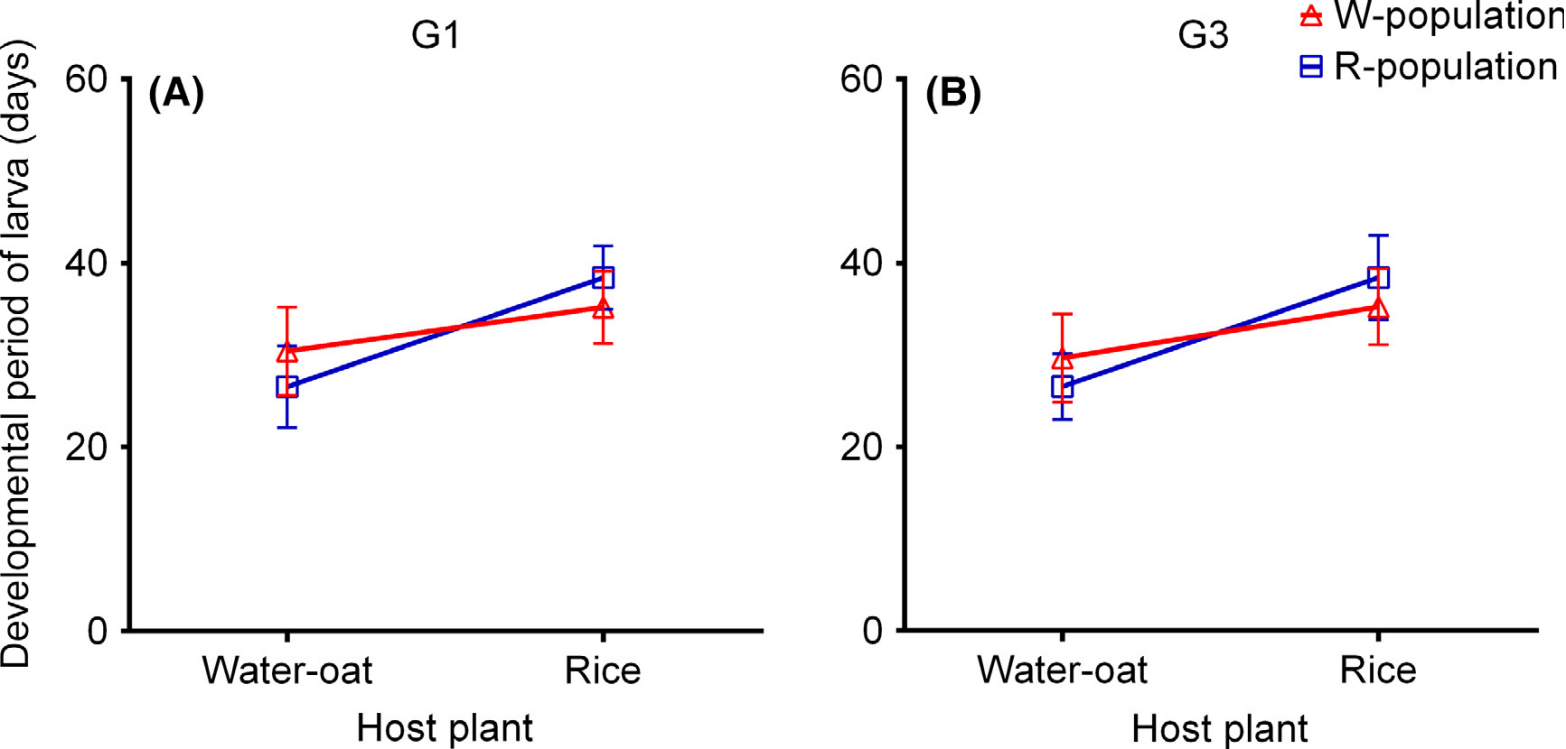
Larval developmental periods of (A) the G1, and (B) G3 generations, of putative rice (R) and water‐oat (W) host races of *Chilo suppressalis* fed on either water‐oats or rice. □ = R, ▵ = W (see Table 1 for statistical analysis).

Larval development was significantly affected by host plant but was not by population origin (Table 1). A significant population–host interaction was, however, apparent between the first and third generation (Table 1). W larvae developed more slowly on rice plants (Tukey's HSD: G1, *P *<* *0.05; G3, *P *<* *0.05) whereas R larvae developed faster on water‐oats (Tukey's HSD: G1, *P *<* *0.05; G3, *P *<* *0.05) (Fig. 3A,B). There were also significant differences in larval development between the putative host races when these were reared on the same host plant (Tukey's HSD: G1, *P *<* *0.05; G3, *P *<* *0.05).

### Effect of host plant on body size

**Figure 4:**
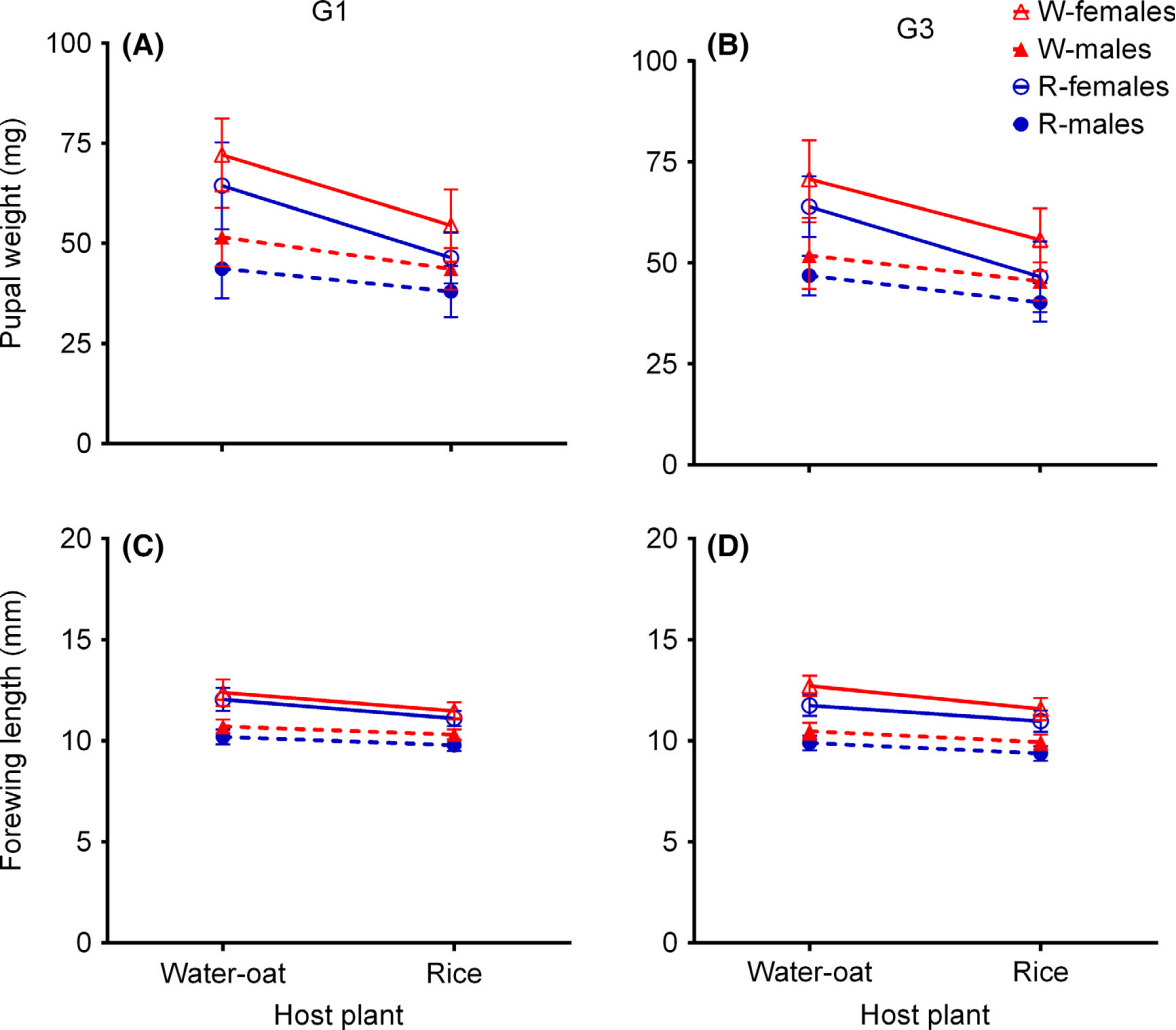
Body size of putative rice (R) and water‐oat (W) host races of *Chilo suppressalis* fed on either water‐oats or rice. (A) Pupal weight of the G1 generation, (B) pupal weight of the G3 generation, (C) forewing length of the G1 generation, and (D) forewing length of the G3 generation (see Table 1 for detailed statistical analysis).

Two indices of body size, pupal weight and adult forewing length, were significantly affected by both putative host race and host plant (Table 1). However, with the exception of the forewing length of G3 females, no significant population–host interaction was apparent in either index (Table 1). In general, the pupal weight (Tukey's HSD: G1, *P *<* *0.05; G3, *P *<* *0.05) and adult forewing length (Tukey's HSD: G1, *P *<* *0.05; G3, *P *<* *0.05) of the W‐r group decreased significantly, and that of the R‐w group increased significantly, with respect to the relevant control groups (Fig. 4A–D). When reared on the same host plant, the pupal weight (Tukey's HSD: G1, *P *<* *0.05; G3, *P *<* *0.05) and adult forewing length (Tukey's HSD: G1, *P *<* *0.05; G3, *P *<* *0.05) of R individuals were less than those of W individuals (Fig. 4A–D).

### Timing of peak mating activity

**Figure 5:**
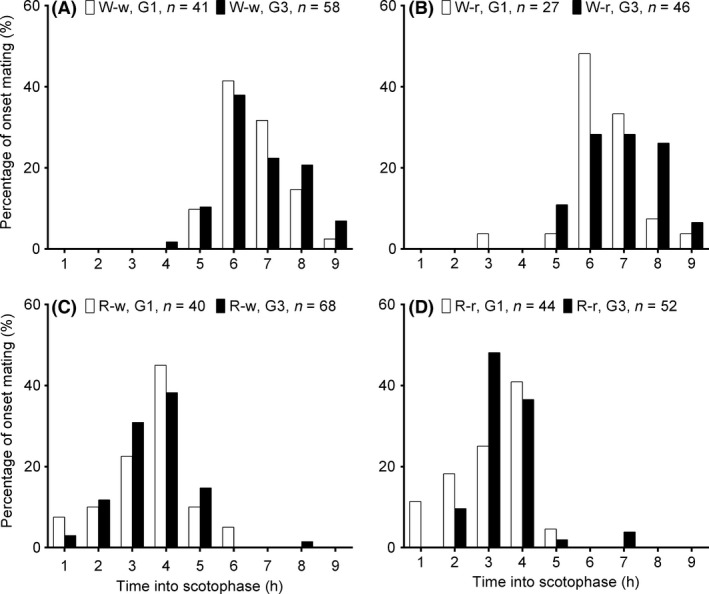
Timing of peak of mating activity of the G1 andG3 generations of putative rice (R) and water‐oat (W) host races of *Chilo suppressalis* reared on either water‐oats (w) or rice (r) (see Fig. 1 for details of the experimental design and Table 2 for statistical analysis).

The peak of mating occurred during the scotophase, ranging from 6 to 7 h in the W‐w and W‐r (Fig. 5A,B), and from 3 to 4 h in the R‐w and R‐r group (Fig. 5C,D). Similar temporal distribution of peak mating activity was observed in both the G1 and G3 generations of these treatment groups (Fig. 5A–D). Therefore, there was *P* a significant difference between two populations on the timing of mating, irrespective of the host plant larvae were reared on (Table 2).

**Table 2:** Results of a chi‐square test of differences in the proportion of individuals engaged in mating activity in G1 and G3 generations of putative rice (R) and water‐oat (W) host races of *Chilo suppressalis* reared on either water‐oats (w) or rice (r) (see Fig. 1 for details of the experimental design). Values in bold are significant at *P *<* *0.05

Generation	Treatment group comparison	Chi‐square	df	*P*‐value
G1	W‐w vs. W‐r	0.328	1	0.567
	R‐w vs. R‐r	3.255	1	0.071
	W‐w vs. R‐w	50.590	1	**0.000**
	W‐r vs. R‐r	49.030	1	**0.000**
	W‐w vs. R‐r	63.170	1	**0.000**
G3	W‐w vs. W‐r	0.670	1	0.413
	R‐w vs. R‐r	0.331	1	0.565
	W‐w vs. R‐w	82.390	1	**0.000**
	W‐r vs. R‐r	71.690	1	**0.000**
	W‐w vs. R‐r	76.410	1	**0.000**

## Discussion

Previous studies of herbivorous insects suggest that adaptation to different host plants can lead to sufficient differences in life‐history traits to cause the reproductive isolation of sympatric host races (Feder and Filchak 1999; Dres and Mallet 2002; Funk et al. 2002). However, distinguishing host races solely on the basis of differences in life‐history traits is unreliable because these may reflect phenotypic plasticity, rather genetic divergence. The results of this study indicate that although the larval survival rate, larval developmental period, and body size of *C. suppressalis* differ significantly between putative rice and water‐oat host races, variation in these traits is also affected by the host plant larvae are reared on. The timing of the peak of mating activity was, however, significantly different in putative host races, irrespective of the host plant larvae were fed on. These results support the hypothesis that observed differences in life‐history traits and the timing of peak mating activity between water‐oat and the rice populations of *C. suppressalis* have a genetic basis. The fact that life‐history traits were affected by host plant indicates that there is a degree of phenotypic plasticity in these parameters but no such phenotypic flexibility was apparent in timing of peak mating activity.

Insect host races typically have higher survival on their usual host plant compared to alternative host plants (Calcagno et al. 2007; Torres‐Vila and Rodríguez‐Molina 2013). However, in this study, both populations of *C. suppressalis* had higher larval survival when reared on water‐oats than when reared on rice. This phenomenon has also been reported in *Rhagoletis pomonella*, in which both the apple race and hawthorn race had higher survival when reared on hawthorn than apple (Prokopy et al. 1988). Differences in the nutritional quality between water‐oat and rice can contribute to the difference in survival rate of *C. suppressalis*. Although the strong host plant effect on larval survival indicates a high degree of phenotypic plasticity with respect to host plant, the significant difference in larval survival between the two putative host races when reared on water‐oats indicates some genetic divergence between with respect to this trait.

The developmental period of insects is a rapidly evolving life‐history trait that plays a crucial role in the synchronization of seasonal life cycles and adaptation to environmental change. Synchronizing development with host plant phenology contributes to host race formation in insects (Feder and Filchak 1999; Ragland et al. 2011). The results of this study indicate that the developmental period of *C. suppressalis* larvae reared on water‐oats was significantly shorter than that of those reared on rice, irrespective of their population of origin. These results are consistent with the seasonal allochrony apparent in the timing of the emergence of *C. suppressalis* adults from rice and water‐oat fields, a phenomenon which has been regarded as evidence of host race formation in this species (Ueno et al. 2006; Matsukura et al. 2009). In addition, because adults are able to migrate between different host plants (Ueno et al. 2006), the significant host plant effect on developmental period may explain the broad overlap in the timing of adult emergence between populations (Maki and Yamashita 1956; Tsuchida and Ichihashi 1995; Ueno et al. 2006; Matsukura et al. 2009). The developmental period of the offspring of such migratory adults would vary according to the host plant they were reared on. The significant differences in developmental period between larvae from different populations reared on the same host plant, and the significant population–host plant interaction effect suggests that there is a genetic component to larval developmental period between populations.

Divergence in body size through adaptation to different hosts can lead to host race formation (Nosil et al. 2002; Hood et al. 2012), and ultimately speciation, in some insects (Diegisser et al. 2004; Ragland et al. 2012). We found that both host plant and putative host race significantly affected both pupal weight and adult forewing length; the W‐r and R‐w groups were smaller in both these indices of body size than their respective W‐w and R‐r control groups. There was, however, considerable overlap between the frequency distributions of pupal weight and forewing length in both putative host races. These results are consistent with previous studies (Maki and Yamashita 1956; Xiao et al. 2006) that indicate that body size in *C. suppressalis* is a population‐specific trait that is also affected by host plant. We suspect that there is more between‐population overlap in body size when adult *C. suppressalis* are able to migrate between different host plants (Ueno et al. 2006). This provides a possible explanation for the anomalous results obtained by a previous study in which body size was used to distinguish the water‐oat from the rice population of *C. suppressalis* (Yu et al. 2002; Matsukura et al. 2006; Ueno et al. 2006; Qian et al. 2007). Nutritional differences between host plants are known to influence both larval growth and nutrient reserves in polyphagous insects, resulting in the differences in body size (Hunter and McNeil 1997; Liu et al. 2007). It is possible, therefore, that *C. suppressalis* may develop quicker, and grow larger, on water‐oats than rice. However, our results show that variation in body size in *C. suppressalis* is the result of both phenotypic plasticity to different host plants and genetic divergence between host races.

Differences in the timing of reproductive activity between closely related insect species or host races have been rarely reported in past decades (Pashley et al. 1992; Konno and Tanaka 1996; Monti et al. 1997). Mating behavior is a circadian activity, and divergence in the timing of mating between populations can lead to allochronic isolation, and ultimately the reproductive isolation, of host‐associated populations of some herbivorous insects (Konno et al. 1981; Pashley et al. 1992). Consistent with the results of previous studies (Ishiguro et al. 2006; Ueno et al. 2006), we found that, irrespective of the species of host plant larvae were reared on, *C. suppressalis* descended from individuals collected in rice fields mated during the first half of the scotophase whereas those descended from individuals collected in water‐oat fields mated during the second half of the scotophase. Although mating behavior can be affected by rearing substrates in *Drosophila* (Havens and Etges 2013), previous work (Ishiguro et al. 2006), and the results of this study, demonstrates that host plant species does not affect the timing of peak mating activity in *C. suppressalis*. These results indicate that the timing of mating activity in *C. suppressalis* is a stable, intrinsic genetic trait that does not change with host plant. We agree with the points that the rice population and water‐oat population have difference in genetics controlling the mating time (Samudra et al. 2002). Divergence in the timing of peak mating activity is, therefore, an allochronic reproductive barrier between the water‐oat and rice populations of *C. suppressalis* that can reliably identify these host races.

In general, the existence of distinct host races in plant‐feeding insects is indicative of adaptation to specific host plants, and conversely, maladaptation to other host plants (Dres and Mallet 2002; Calcagno et al. 2007). However, the growth and survival of both putative host races of *C. suppressalis* were higher when larvae were reared on water‐oats than on rice. A similar finding has also been reported in *R. pomonella*, in which the apple race had higher survival from the egg to pupal stage when reared on hawthorn (Prokopy et al. 1988), and in *Lobesia botrana* in which larval survival, adult weight, and development, improved on an alternate host (Torres‐Vila and Rodríguez‐Molina 2013). For *R. pomonella* and *L. botrana*, these improvements could be related to nutritional differences between host plants, the history of these plant species as alternative hosts for these insects, and their agroecological context (i.e., introduction and cultivation) (Prokopy et al. 1988; Torres‐Vila and Rodríguez‐Molina 2013). It is possible that the better growth and survival of *C. suppressalis* on water‐oats than on rice reflects differences in the nutritional quality of these host plants. Water‐oats are probably a less‐utilized resource for *C. suppressalis* than rice. It is unclear which host plant the ancestral host is.

Different host plant preferences during the larval or adult stage can lead to phenotypic plasticity in mating signals in phytophagous insects, and ultimately the behavioral isolation of host‐associated populations (Landolt and Phillips 1997; Geiselhardt et al. 2012; Havens and Etges 2013; Etges and de Oliveira 2014). However, the results of this study show that the timing of peak mating activity in *C. suppressalis* is a population characteristic independent of host plant. Divergence in peak mating time could cause the temporal reproductive isolation of host‐associated populations of this species (Samudra et al. 2002; Ueno et al. 2006). Since the timing of mating activity in some insects is controlled by an endogenous clock (Sakai and Ishida 2001; Miyatake et al. 2002), it is possible that the observed differences in this trait between the host races of *C. suppressalis* reflect divergence in the genes that control this clock. Although some of the observed between‐population variation in life‐history traits can be attributed to phenotypic plasticity, a component of this variation appears to have a genetic basis. The results of this study, therefore, support the existence of host races in *C. suppressalis*. More geographical replication is, however, required to confirm that the observed differences in life‐history traits and timing of peak mating activity reflect adaptation to different host plants, rather than other factors, such as local climate, parasite pressure, and soil type.

In conclusion, the results of this study suggest that differences in some life‐history traits and the timing of peak mating activity between host‐associated populations of *C. suppressalis* have a genetic basis. Although life‐history traits were influenced by both genetic and host plant effects, differences in the timing of peak mating activity between host races were not affected by host plant and therefore are indicative of genetic divergence between host races. Divergence in the timing of mating activity is an important potential mechanisms for the development of reproductive isolation between sympatric populations in this species. Further research is required to investigate the physiological and genetic mechanisms regulating the timing of mating activity in host races of *C. suppressalis*. Such research could provide insights into how allochronic mating behavior evolves in the wild.

## Conflict of Interest

None declared.
